# Aspirin ameliorates the long‐term adverse effects of doxorubicin through suppression of cellular senescence

**DOI:** 10.1096/fba.2019-00041

**Published:** 2019-09-09

**Authors:** Mingxiao Feng, Joohwee Kim, Kevin Field, Christine Reid, Ioulia Chatzistamou, Minsub Shim

**Affiliations:** ^1^ Department of Biological Sciences University of South Carolina Columbia SC USA; ^2^ Center for Colon Cancer Research University of South Carolina Columbia SC USA; ^3^ UNC School of Medicine University of North Carolina at Chapel Hill Chapel Hill NC USA; ^4^ Department of Pathology, Microbiology & Immunology School of Medicine University of South Carolina Columbia SC USA; ^5^ Department of Biochemistry College of Graduate Studies and Arizona College of Osteopathic Medicine Midwestern University Glendale AZ USA

**Keywords:** chemotherapy, cyclooxygenase, senescence

## Abstract

A number of childhood cancer survivors develop adverse, late onset side effects of earlier cancer treatments, known as the late effects of cancer therapy. As the number of survivors continues to increase, this growing population is at increased risk for a number of health‐related problems. In the present study, we have examined the effect of aspirin on the late effects of chemotherapy by treating juvenile mice with doxorubicin (DOX). This novel mouse model produced various long‐term adverse effects, some of which resemble premature aging phenotypes. DOX also resulted in the tissue accumulation of senescent cells and up‐regulation of cyclooxygenase‐2 (COX2) expression. However, treatment with aspirin following juvenile exposure to DOX improved body weight gain, ameliorated the long‐term adverse effects, and reduced the levels of senescence markers. Moreover, aspirin reduced p53 and p21 accumulation in DOX‐treated human and mouse fibroblasts. However, the suppressive effect of aspirin on DOX‐induced p53 accumulation was significantly decreased in COX2 knockout mouse embryonic fibroblasts. Additionally, treatment of senescent fibroblasts with aspirin or celecoxib, a COX2 specific inhibitor, reduced cell viability and decreased the levels of Bcl‐xL protein. Taken together, these studies suggest that aspirin may be able to reduce the late effects of chemotherapy through the suppression of cellular senescence.

**Abbreviations**
COX2: cyclooxygenase-2
DOX: doxorubicin
NSAIDs: non-steroidal anti-inflammatory drugs

## 1 INTRODUCTION

Survivors of childhood cancer often experience the late effects of cancer therapy including cardiovascular and respiratory diseases, musculoskeletal complications, infertility, endocrine and metabolic disorders, premature skin and ocular changes, early onset of frailty, and cognitive problems, which manifest months to years after completion of cancer treatment.^1^ Current estimates indicate that there are over 400 000 survivors of childhood cancer living in the United States.^2^ Given that the survival rates are improving due to advances in treatment, this growing population is at increased risk for a number of chronic or life‐threating health conditions. However, our understanding of the molecular and/or cellular mechanisms that underlie the development of cancer therapy‐induced adverse sequelae is limited.

One of the possible mechanisms by which these long‐term side effects may manifest is the increased occurrence of cellular senescence. It is well‐described that DNA damaging agents including radiation and chemotherapy drugs induce senescence in cultured cells.^3^, ^4^ In addition, it has been shown that chemotherapy induces cellular senescence in human patients.^5^ Moreover, two recent studies have shown that the selective removal of chemotherapy‐induced senescent cells ameliorates adverse effects of chemotherapy and improves tissue homeostasis in mice.^6^, ^7^ Senescent cells increase with age in mammalian tissues,^8^ and have been found at sites of age‐related pathologies such as osteoarthritis and atherosclerosis,^9^, ^10^ suggesting that senescence plays a role in organismal aging. Consistent with the role of senescence in aging, the survivors of childhood cancer are also often associated with an early occurrence of health conditions related to aging such as neurocognitive decline, metabolic disorders, musculoskeletal complications, premature skin and ocular changes, and early onset of frailty.^11^

Nonsteroidal anti‐inflammatory drugs (NSAIDs) which inhibit cyclooxygenase (COX) enzymes are one of the most commonly consumed drugs in the world. Clinical studies have shown that aspirin, a classical NSAID, has a chemopreventive effect against colorectal cancer, in which aging is the major risk factor.^12^ Aspirin has also been reported to have therapeutic and/or preventive effects on other human age‐related diseases including atherosclerosis, arthritis, osteoporosis, and Alzheimer's disease.^13^, ^14^, ^15^ Given the strong association between senescence and aging, the mechanism underlying beneficial effects of aspirin against age‐related diseases may be mediated through suppression of cellular senescence. However, the effect of aspirin on cellular senescence has not been clearly determined.

In the current study, we utilized a novel mouse model to examine the ability of aspirin to suppress senescence induced by the chemotherapeutic drug doxorubicin (DOX). We found that early treatment of juvenile mice with DOX, one of the most widely used chemotherapeutics for childhood cancers,^16^, ^17^ causes tissue accumulation of senescent cells and development of adverse effects in adult mice. We also found that aspirin significantly reduces senescent cell accumulation and improves late effects in DOX‐treated mice.

## 2 MATERIALS AND METHODS

### 2.1 Materials

p53 (#2524), Bcl‐xL (#2762), and GAPDH (#2118) antibodies were purchased from Cell Signaling Technology. COX2 (#160106) antibody was purchased from Cayman Chemical. Doxorubicin was purchased from Sigma. Aspirin and celecoxib were purchased from Cayman Chemical.

### 2.2 Cell lines and treatment

Cells were cultured in Dulbecco's Modified Eagle Medium (DMEM) (Corning) supplemented with 10% fetal bovine serum (FBS) (HyClone) and gentamicin (Sigma‐Aldrich). Mouse embryonic fibroblasts (MEFs) were prepared from E13.5 wild‐type and COX2 knockout embryos using a standard protocol.^18^ MRC5 human fibroblasts were obtained from the American Type Culture Collection (ATCC). For treatment with DOX, 2.5 × 10^5^ cells were plated in a 60 mm cell culture dish and 48 hours later, the cells were pretreated with the aspirin for 1 hour and then co‐treated with aspirin for 24 hours. Senescence of cultured cells was induced by incubation with 0.25 μmol/L DOX for 24 hours.^6^

### 2.3 Mice and treatment

All animal studies and procedures were approved by the University of South Carolina Institutional Animal Care and Use Committee. C57BL/6 male and female mice used in this study were obtained from the Jackson Laboratory. For DOX treatments, 2‐week‐old mice were injected four times (once weekly for 4 consecutive weeks) intraperitoneally with PBS or DOX (1.25 mg/kg in saline). For aspirin treatment, 9‐week‐old, PBS‐ or DOX‐treated mice were fed aspirin (0.02 g/100 ml in drinking water) for 7‐9 weeks. For glucose tolerance test, mice were fasted overnight and intraperitoneally injected with D‐glucose (2 g/kg). Blood glucose was measured 0, 30, 60, and 120 minutes after glucose injection using AlphaTRAK2 glucometer and test strips. White blood cell (WBC) counting was carried out with VetScan HM5 Hematology System (Abaxis). Blood for blood cell counting was sampled from the mouse by retro‐orbital collection.

### 2.4 Viability assay

For simultaneous observation of viable and nonviable cells, cells were stained with Calcein AM and DAPI according to our previously reported protocol.^19^ Briefly, both the adherent and detached cells were collected and incubated in PBS containing Calcein AM (0.2 µmol/L) and DAPI (1 µg/mL) for 10 minutes at 37°C. The number of live and dead cells was counted under a fluorescent microscope and the percentage of live cells was calculated as cell viability.

### 2.5 Quantitative RT‐PCR

RNA was isolated using an RNeasy kit (Qiagen) and treated with 1 unit of amplification grade DNase I (Invitrogen) per 1 µg RNA at room temperature for 15 minutes to remove genomic DNA followed by the inactivation of the DNase I with 2.5 mmol/L EDTA (pH 8.0) and incubation at 65°C for 5 minutes. Reverse transcription was performed with 2 µg total RNA using the SuperScript II reverse transcription system (Invitrogen) according to the manufacturer's instructions. Quantitative real‐time PCR analysis was conducted using a GoTaq® qPCR mixture (Promega). The primer pairs used in this study include: p16 (5′‐GAACTCTTTCGGTCGTACCC‐3′ and 5′‐CGAATCTGCACCGTAGTTGA‐3′), p21 (5′‐TTGTCGCTGTCTTGCACTCT‐3′ and 5′‐TTTCGGCCCTGAGATGTTCC‐3′), IL6 (5′‐TTGGGACTGATGCTGGTGAC‐3′ and 5′‐ CTGTGAAGTCTCCTCTCCGG‐3′), and GAPDH (5′‐GGACCTCATGGCCTACATGG‐3′ and 5′‐TAGGGCCTCTCTTGCTCAGT‐3′). Amplification and detection of specific mRNAs were performed using Eppendorf RealPlex^2^. Reactions were run in triplicate for three independent experiments. The mean of housekeeping gene GAPDH was used as an internal control to normalize the variability in expression levels. Expression data were normalized to the mean of GAPDH to control the variability in expression levels and were analyzed using the 2^−ΔΔCT^ method.

### 2.6 Western blot analysis

Cell and tissue lysates were prepared with ice‐cold RIPA buffer (50 mmol/L Tris–HCl, pH 7.4, 1% NP‐40, 0.5% Na‐deoxycholate, 0.1% SDS, 1 mmol/L Na3VO4, 10 mmol/L NaPPi, 10 mmol/L beta‐glycerophosphate and 50 mmol/L NaF) supplemented with the Halt protease inhibitor cocktail (Thermo Scientific). Protein concentration was measured using the BCA protein assay kit (Pierce). Equal amounts of protein were solubilized and heated at 75°C in LDS sample buffer (Invitrogen) with sample reducing agent (Invitrogen) for 10 minutes, and then separated by SDS‐PAGE, and transferred to Immobilon‐P membrane (Millipore). Following incubation in blocking buffer (TBS with 5% nonfat dry milk and 0.1% Tween 20) for 1 hour at room temperature, the membranes were probed overnight at 4°C. The membranes were washed and then probed with an HRP‐linked secondary antibody (Cell Signaling Technology) for 1 hour at room temperature. Specific proteins were detected using enhanced chemiluminescence (ECL) Western blotting detection kit (GE Healthcare) according to the manufacturer's instructions. All western blot analysis was repeated for at least three times.

### 2.7 Histology and senescence‐associated beta‐galactosidase (SA‐β‐gal) staining

Mouse tissues were collected, fixed in 10% neutral buffered formalin, and embedded in paraffin blocks. Tissue sections (5 µm) were subjected to hematoxylin‐eosin (H&E) staining by standard procedures. After mounting, the sections were observed under an Olympus BX51 light microscope, and the image was acquired by an AxioCam MRc camera. Cross‐sectional area (CSA) of the skeletal muscle fiber, the thickness of subcutaneous fat, and the sizes of adipocytes were measured with ImageJ software (National Institutes of Health). SA‐β‐gal staining was carried out with a modified protocol from Dimiri et al.^20^ Briefly, frozen sections were first rinsed with PBS and fixed in a fixative solution (0.2% glutaraldehyde, 0.185% formaldehyde in PBS) for 3 minutes. Sections were then rinsed twice with PBS and stained in staining solution [1 mg/mL 5‐bromo‐4‐chloro‐3‐indolyl‐β‐D‐galactopyranoside (X‐gal), 40 mmol/L citric acid sodium phosphate (pH 6.0), 150 mmol/L NaCl, 2 mmol/L MgCl_2_, 5 mmol/L potassium ferrocyanide, 5 mmol/L potassium ferricyanide] in dark at 37°C for 2 hours ~overnight.

### 2.8 Statistical analysis

Data are expressed as the mean ± SEM of at least three independent experiments. The statistical analysis was done using a Student's t‐test or One‐way ANOVA, using GraphPad Prism 5 software (GraphPad Software). Statistical significance is indicated by asterisks in figures: * for *P* values <.05, ** for *P* values <.01, and *** for *P* values <.001.

## 3 RESULTS

### 3.1 Treatment of juvenile mice with doxorubicin results in development of long‐term adverse effects in adults

**Figure 1:**
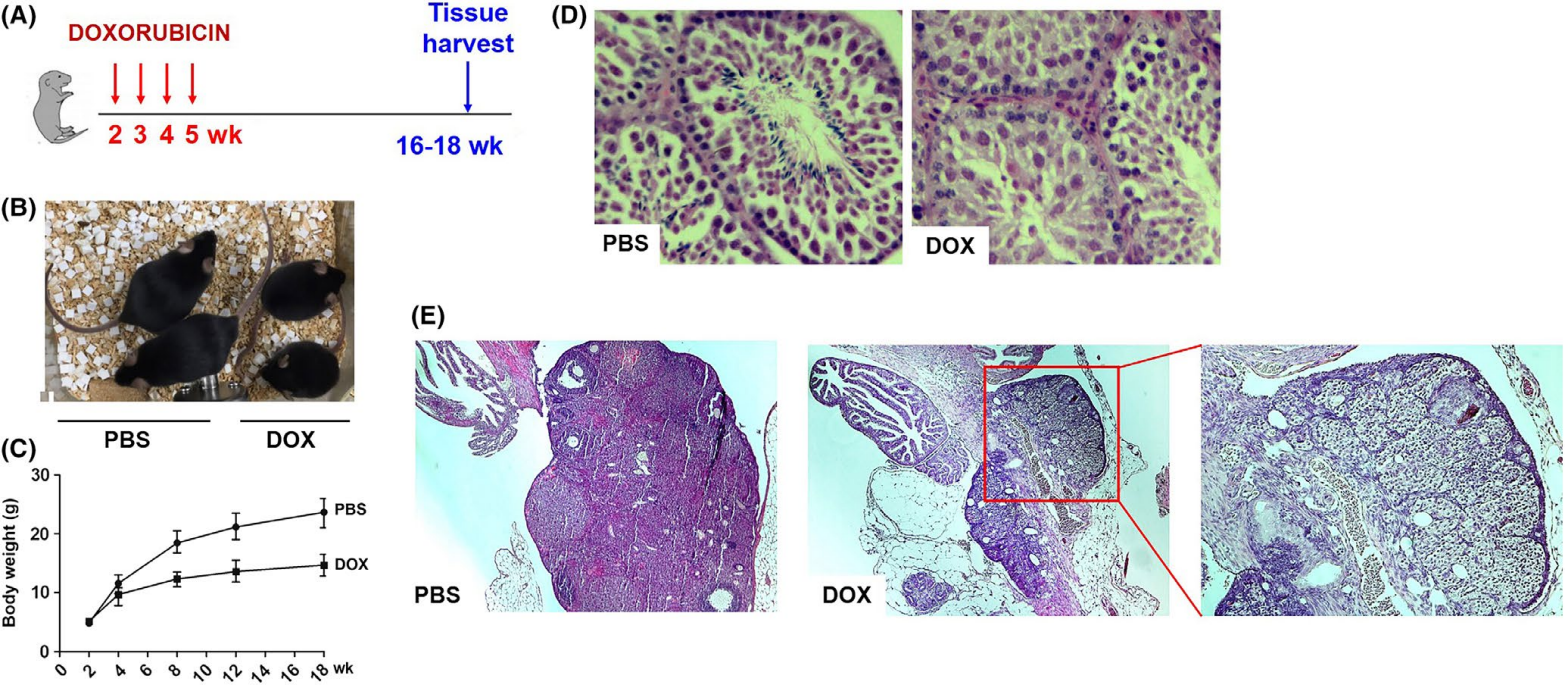
A, Experimental scheme for doxorubicin (DOX) treatment of C57BL/6 mice. B, A representative photo of 12‐wk‐old mice injected with PBS or DOX at 2 wk of age. C, Body weight gain in PBS or DOX‐treated mice (n = 6). D and E, Cross‐sections of the testis (D) and ovary (E) in 18‐wk‐old PBS or DOX‐treated mice at 2 wk of age

**Figure 2:**
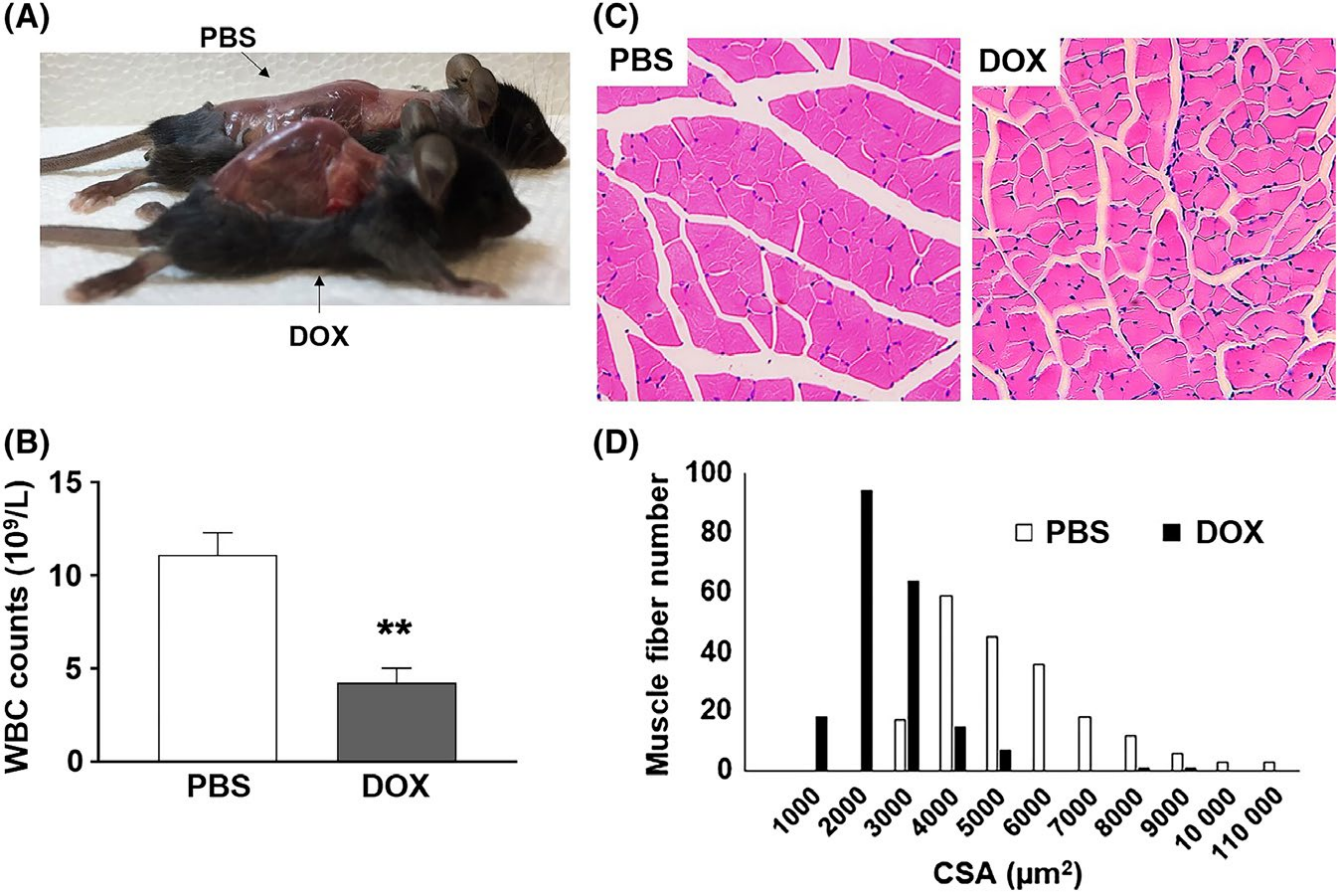
A, A photograph of skinned PBS‐ or DOX‐treated mouse that exhibits pronounced kyphosis. B, White blood cell (WBC) counts in 16‐18‐wk‐old PBS‐ or DOX‐treated female C57BL/6 mice (n = 4, **P < .01). C, H&E‐stained sections of hind leg skeletal muscle from 18‐wk‐old PBS‐ and DOX‐treated female littermates. D, Frequency distribution of skeletal muscle fiber cross‐sectional area (CSA) between PBS‐ and DOX‐treated mice

The anthracyclines, including DOX, are among the most widely used and successful chemotherapeutics for childhood cancers. Mouse models have been used to study DOX‐induced chronic or acute cardiotoxicity.^21^, ^22^, ^23^, ^24^ However, these models use adult mice (6‐12 weeks old), and DOX‐treated mice often die due to complications in cardiac function, which make them inappropriate to model the long‐term side effects of chemotherapy in childhood cancer survivors. To investigate the late effects of chemotherapy for childhood cancer, we developed a new mouse model. As shown in Figures 1A, 2‐week‐old C57BL/6 mice have received four intraperitoneal injections of a low‐dose DOX (1.25 mg/kg in saline) at 7‐day intervals. Eleven to 13 weeks later, the phenotypes of the treated mice (16‐ to 18‐week‐old) were analyzed.

Slowed growth and impaired sexual development are common problems associated with childhood cancer therapy. As shown in Figure 1B,C, DOX‐treated mice exhibited reduced growth and body weight gain. The negative effect of DOX on the body weight gain was observed during DOX treatment (week 4 in Figure 1C) and became more significant as the mice aged. Histological analysis of the testes showed that there were all different stages of differentiation of the seminiferous epithelium, including spermatogonia, spermatocytes, round spermatids, and elongated spermatids in the seminiferous tubules of the PBS‐treated animals. In contrast, only a few elongated spermatids were observed in the seminiferous epithelium of the 16‐ to 18‐week‐old DOX‐treated animals, suggesting that DOX‐treated animals may be infertile due to defects in spermatogenesis (Figure 1D). In addition, the ovaries from 16‐ to 18‐week‐old DOX‐treated animals were generally smaller in size and exhibited a considerable reduction of primary, secondary, and antral follicles (Figure 1E). To further analyze the effect of DOX on reproductive function, we set up matings between four DOX‐treated male and four control female mice. However, none of these female mice became pregnant after 2 months of being housed together. Similarly, when four female DOX‐treated mice were set in the same cage with four male control mice of the same age, only one out of these four females was found pregnant, indicating that DOX treatment causes a decline in fertility.

**Figure 3:**
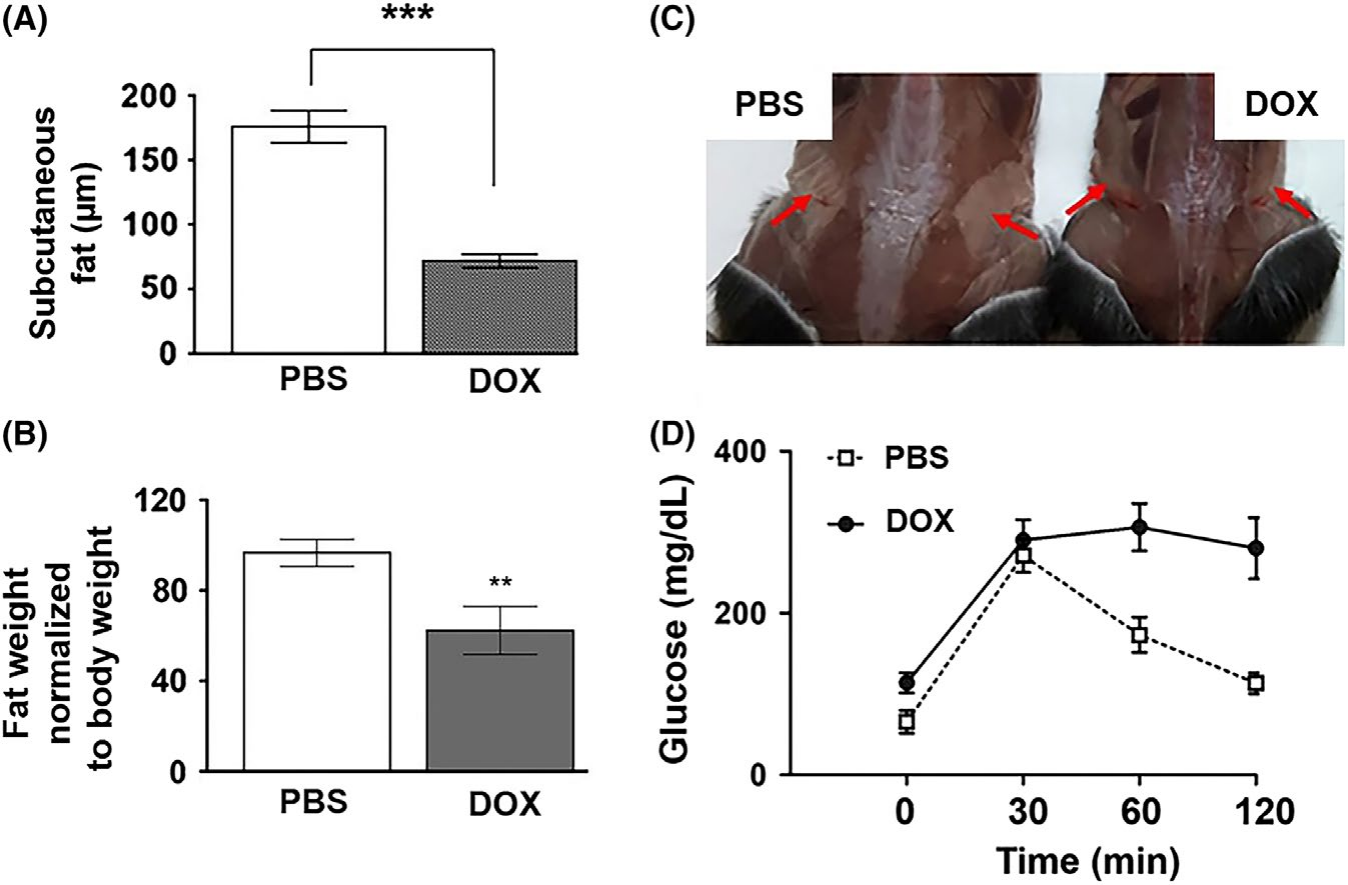
A, Quantification of the thickness of subcutaneous fat in 16‐18‐wk‐old mice treated with PBS or DOX at 2 wk of age (n = 4, ***P < .001). B, The weight of inguinal fat normalized to body weight in PBS‐ or DOX‐treated mice (n = 4, **P < .01). C, A representative photo of inguinal fat (arrows) in 17‐wk‐old PBS or DOX‐treated mice. D, Glucose tolerance test in mice injected with PBS or DOX at 2 wk of age (n = 4)

The 16‐ to 18‐week‐old adult mice treated with DOX at their juvenile stage also exhibited phenotypes characteristic of aging. Kyphosis (6 out of 22, Figure 2A) and corneal opacity (three out 22, data not shown) were observed in DOX‐treated animals. Additionally, white blood cell (WBC) counts in DOX‐treated animals were significantly lower than those in saline‐injected control littermates (Figure 2B). The size of muscle fiber is known to decrease with increasing age.^25^ As shown in Figure 2C,D, the cross‐sectional area of skeletal muscle fiber in the hind leg of DOX‐treated mice was significantly decreased compared to that in PBS‐injected control littermates. The reduced thickness of subcutaneous fat, one of the markers for aged skin,^26^ was observed in DOX‐treated mice (Figure 3A). In addition to subcutaneous fat, fat tissue mass is known to decline in old age.^27^ Consistent with this, DOX‐treated animals displayed decreased body fat mass and impaired glucose tolerance (Figure 3B‐D).

### 3.2 Increased senescence in the tissues of mice treated with DOX at the juvenile stage

**Figure 4:**
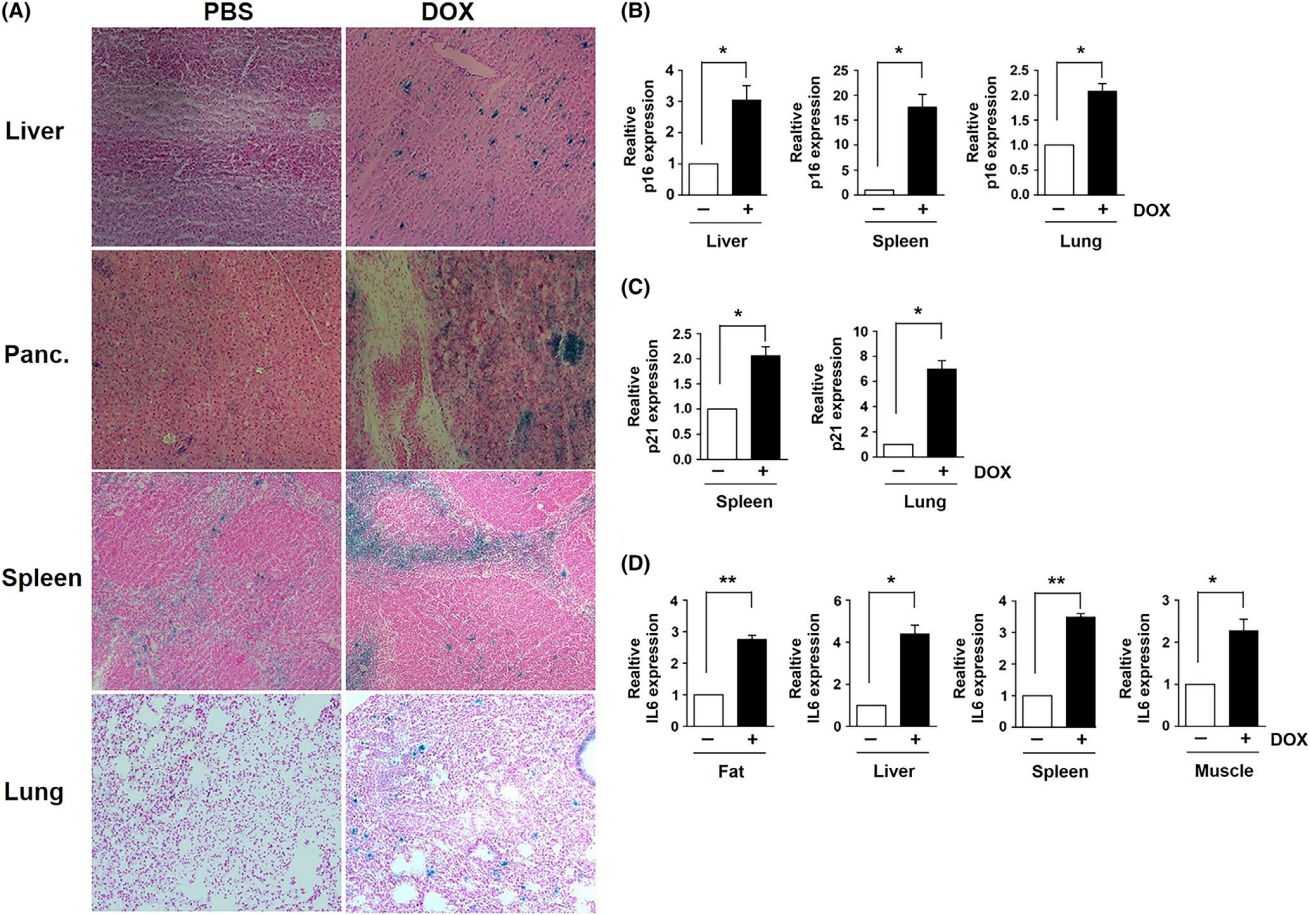
A, Images of SA‐β‐gal staining in the tissues of 16‐ to 18‐week‐old mice treated with PBS or DOX at 2 weeks of age (n = 4). mRNA levels of (B) p16, (C) p21, and (D) IL6 in the tissues of PBS and DOX‐treated mice. Results expressed as means ± SEM (n = 4, *P < .05, **P < .01)

Numerous in vitro and in vivo studies have shown that DNA damaging agents including radiation and chemotherapeutic agents induce senescence.^3^, ^4^, ^5^, ^6^, ^7^ In addition, the selective removal of senescent cells has been shown to ameliorate the side effects of DOX in mice.^6^, ^7^ To determine whether cellular senescence plays a role in the long‐term adverse effects that we observed, we analyzed the levels of senescence markers in the tissues of the 16‐ to 18‐week‐old mice treated with DOX in their juvenile stage. Senescent cells are reported to exhibit increased activity of senescence‐associated galactosidase (SA‐β‐gal), a widely used senescence marker.^20^ Senescent cells also have increased expression of cyclin‐dependent kinase inhibitors including p16 and p21. Additionally, senescent cells secrete inflammatory cytokines and extracellular proteases known as the senescence‐associated secretory phenotype (SASP) that are linked to chronic inflammation and disruption of tissue function.^4^, ^28^ To determine the levels of SA‐β‐gal activity, cryosections were made from the tissues of control and DOX‐treated mice, and SA‐β‐gal staining was performed. As shown in Figure 4A, increased numbers of SA‐β‐gal‐positive cells were observed in liver, spleen, pancreas, and lung of DOX‐treated mice when compared to their control littermates. We also measured the mRNA level of the senescence markers including p16, p21, and IL6 in various tissues. The p16 mRNA level was elevated in liver, spleen, and lung of DOX‐treated mice at their juvenile stage (Figure 4B), while p21 mRNA level was increased in spleen and lung (Figure 4C). The levels of IL6 mRNA were increased in the liver, skeletal muscle, spleen, and inguinal fat of DOX‐treated mice compared to those in PBS‐treated mice (Figure 4D).

### 3.3 Aspirin reduces the cellular senescence and long‐term adverse effects in adult mice treated with DOX at the juvenile stage

**Figure 5:**
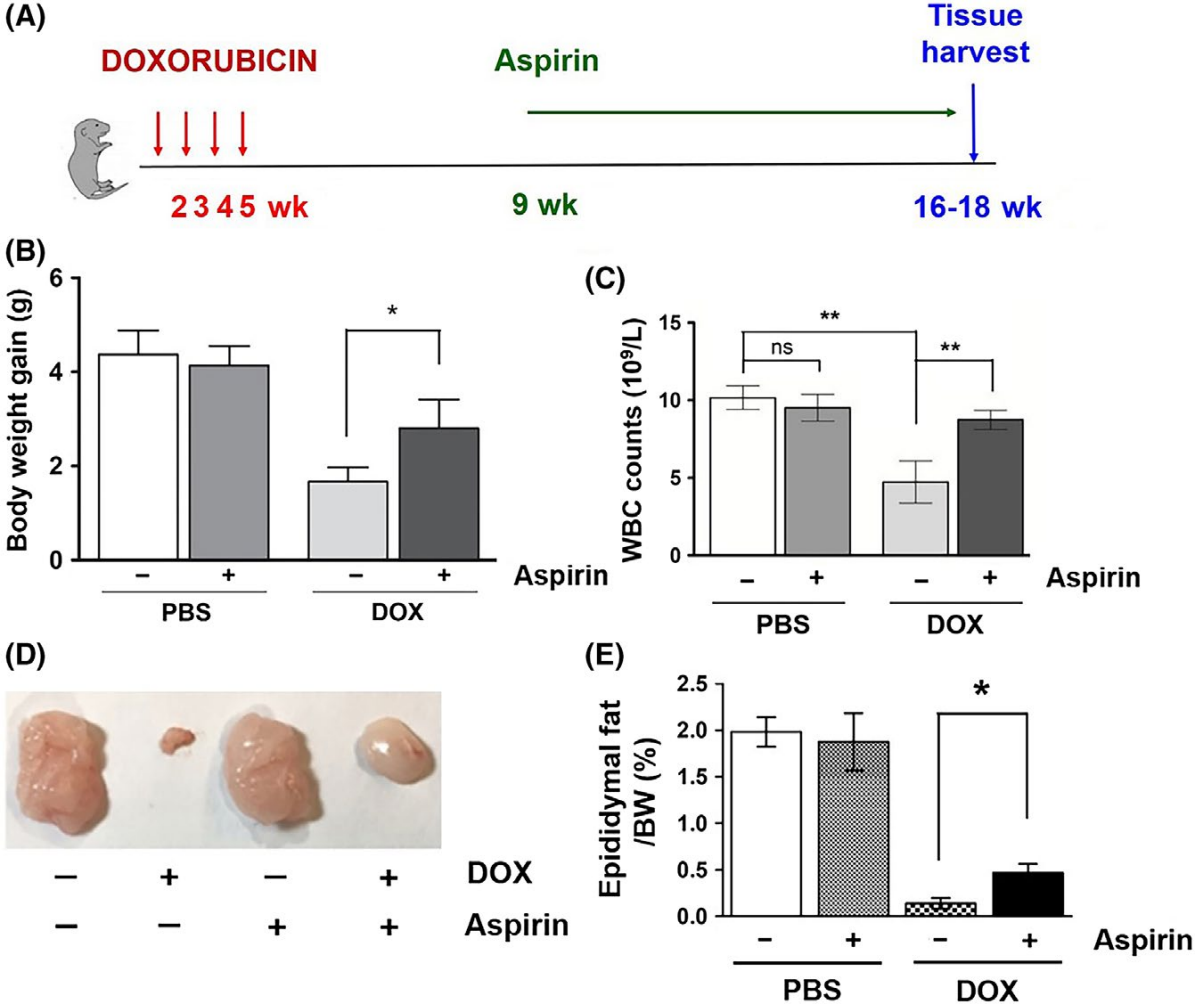
A, Experimental scheme for doxorubicin (DOX) and aspirin treatments. C57BL/6 mice were treated four times, once a week, from 2 wk of age with doxorubicin. One month later, the mice were fed drinking water with or without aspirin (0.02 g/100 mL) for 8 wk. B, Body weight gain and (C) white blood cell counts in PBS, aspirin, DOX, and DOX/aspirin‐treated male mice (n = 5). D, A representative photo of epididymal fat in PBS, aspirin, DOX, and DOX/aspirin‐treated male mice. E, Epididymal fat weight normalized to body weight (n = 4, *P < .05). Results expressed as means ± SEM

**Figure 6:**
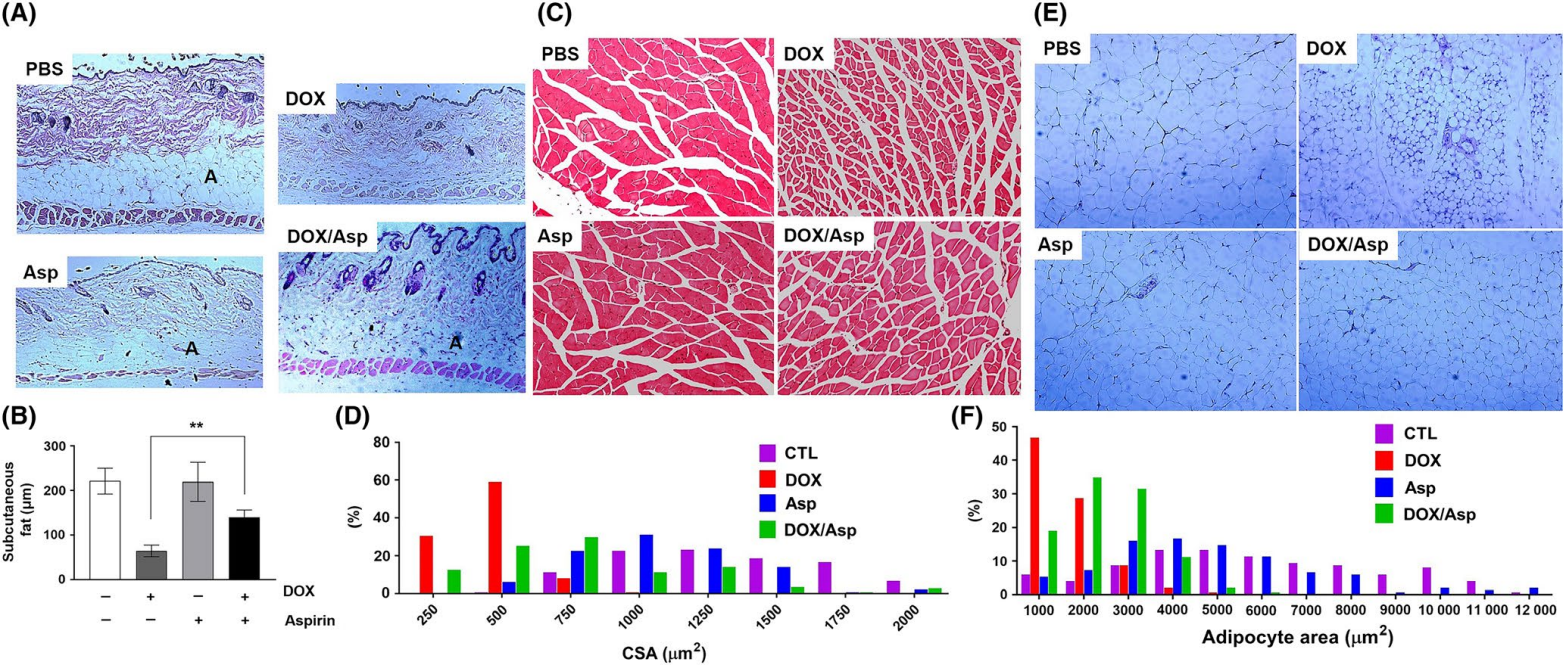
A, Cross‐sections of dorsal skin from 19‐wk‐old PBS, aspirin, DOX, and DOX/aspirin‐treated mice. Adipose tissue under the dermis [A] is indicated. B, Quantification of the thickness of subcutaneous fat (n = 4, **P < .01). C, H&E‐stained sections of hind leg skeletal muscle from PBS, aspirin, DOX, and DOX/aspirin‐treated mice. D, Frequency distribution of skeletal muscle fiber cross‐sectional area (CSA). E, H&E‐stained sections of fat from PBS, aspirin, DOX, and DOX/aspirin‐treated mice. F, Frequency distribution of adipocyte size

As previously described, numerous studies have shown that aspirin has beneficial effects against many human age‐related diseases. Thus, we hypothesized that aspirin may suppress senescence in DOX‐treated animals and improve the long‐term adverse effects of chemotherapy. To test this, 2‐week‐old mice were treated either with PBS or DOX as described earlier. The mice were then allowed to recover for 1 month, fed aspirin (0.02 g/100 mL in drinking water) for 7‐9 weeks, and analyzed for their phenotypes (Figure 5A). As expected, the body weight gain in DOX‐treated mice was significantly reduced compared to that in control mice. However, aspirin treatment significantly improved body weight gain in DOX‐treated mice (Figure 5B). In addition, the reduction in WBC count caused by DOX was partially recovered by aspirin feeding (Figure 5C). Moreover, the loss of fat mass in DOX‐treated mice was also significantly improved by aspirin treatment (Figure 5D,E). Histological analysis of tissue sections further revealed that aspirin significantly ameliorates the loss of subcutaneous fat (Figure 6A,B), the decrease in muscle fiber size (Figure 6C,D), and the reduction in adipocyte size (Figure 6E,F) in DOX‐treated mice.

**Figure 7:**
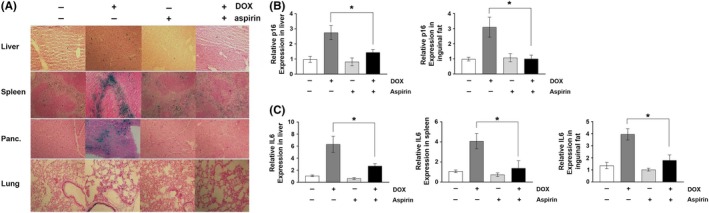
A, Images of SA‐β‐gal staining in the tissues of PBS, aspirin, DOX, and DOX/aspirin‐treated mice (n = 4). mRNA levels of (B) p16 and (C) IL6 in the tissues of PBS, aspirin, DOX, and DOX/aspirin‐treated mice (n = 4, *P < .05)

The fact that aspirin improved DOX‐induced long‐term adverse effects encouraged us to test if aspirin could suppress the cellular senescence induced by DOX. To analyze the effect of aspirin on DOX‐induced senescence in mice, SA‐β‐gal staining was performed with cryosections of liver, spleen, pancreas, and lung, the tissues that exhibit high levels of SA‐β‐gal‐positive cells following DOX treatment. As shown in Figure 7A, aspirin significantly reduced the number of SA‐β‐gal‐positive cells in these tissues. Moreover, DOX‐induced up‐regulation of p16 and IL6 in the liver, inguinal fat, and spleen was also significantly reduced by aspirin treatment (Figure 7B,C).

### 3.4 NSAIDs inhibit the signaling pathways involved in senescence

**Figure 8:**
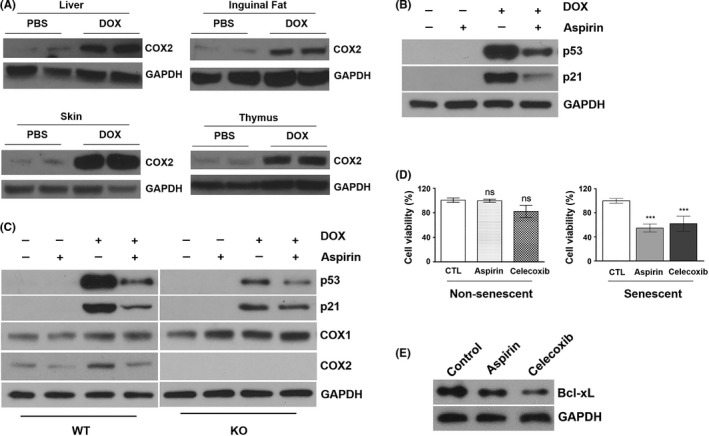
A, Western blot analysis of COX2 and GAPDH in liver, inguinal fat, skin, and thymus of 16‐wk‐old PBS and DOX‐treated mice. B, MRC5 normal human fibroblasts were pretreated with 1 mmol/L aspirin for 1 h and then co‐treated with 0.5 μmol/L doxorubicin for 24 h. Total cell lysates were subjected to western blot analysis for p53, p21, and GAPDH. C, Wild‐type (WT) and COX2 knockout (KO) MEFs were pretreated with 1 mmol/L aspirin for 1 h and then co‐treated with 0.5 μmol/L doxorubicin for 24 h. Total cell lysates were subjected to western blot analysis for p53, p21, COX1, COX2, and GAPDH. D, Nonsenescent and DOX‐induced senescent MRC5 normal human fibroblasts were treated with aspirin (1 mmol/L) or celecoxib (20 µmol/L) (3 d for nonsenescent cells and 12 d for senescent cells, respectively). Treated cells were stained with Calcein AM and DAPI for visualization of live and dead cells, respectively. Data are presented as the percent of live cells. ***P < .001. E, Western blot analysis of Bcl‐xL in senescent MRC5 cells treated with 1 mmol/L aspirin and 20 μmol/L celecoxib for 72 h

Aspirin is thought to exert its effects through inhibition of COX enzymes.^29^ In addition, we previously have reported that the inducible, transgenic expression of COX2 causes accumulation of senescent cells and early aging phenotypes in adult mice.^30^ Therefore, we focused our investigation on the role of COX2 in DOX‐induced cellular senescence. We first analyzed the levels of COX2 in 16‐ to 18‐week‐old mice treated with DOX at their juvenile stage. It is very well‐known that COX2, an inducible isoform of COX, is highly induced by DNA damaging agents including radiation and chemotherapy.^18^, ^31^, ^32^, ^33^ Consistent with this, COX2 expression was up‐regulated in the tissues of DOX‐treated mice (Figure 8A).

To examine the contribution of COX2 to DOX‐induced cellular senescence, we analyzed the signaling pathways known to be involved in cellular senescence. Previously, we have shown that pharmacological inhibition or genetic deletion of COX2, but not COX1, suppresses DOX‐induced p53 accumulation in mouse embryonic fibroblasts (MEFs) and human cancer cell lines.^18^ Since p53 plays an important role in cellular senescence,^34^ we examined whether aspirin suppresses DOX‐induced accumulation of p53 in normal human fibroblasts. As shown in Figure 8B, pretreatment with aspirin significantly reduced p53 protein levels in DOX‐treated MRC5 human fibroblasts. Similarly, the levels of p21, a transcriptional target of p53, were decreased by aspirin pretreatment. To determine whether COX2 plays a role in aspirin's effect on p53, we treated wild‐type (WT) and COX2 knockout (KO) MEFs with DOX in the presence or absence of aspirin. Consistent with our previous results,^18^ DOX‐induced accumulation of p53 protein was decreased in COX2 KO MEFs compared to that in WT MEFs (Figure 8C). However, the suppressive effect of aspirin on p53 was reduced in COX2 KO MEFs compared to WT MEFs, suggesting that aspirin may suppress DOX‐induced p53 accumulation through the inhibition of COX2.

Additionally, we also examined whether aspirin regulates the signaling molecules that are involved in the maintenance of cellular senescence. Numerous studies have demonstrated that NSAIDs induce apoptosis in various human cancer cells.^35^ Proapoptotic mechanisms of NSAIDs include downregulation of Bcl‐2/Bcl‐xL expression, inhibition of PI3K/Akt or NF‐κB signaling, suppression of prostaglandin receptor‐mediated cAMP/PKA signaling, and regulation of p53 family members.^36^, ^37^, ^38^ To test the possibility that aspirin may affect the viability of senescent cells, normal human fibroblasts were first treated with DOX to induce senescence (Supplementary Figure S1), and incubated with aspirin or celecoxib, a COX2‐specific inhibitor, for 12 days. As shown in Figure 8D, the viability of senescent MRC5 cells was decreased by incubation with either aspirin or celecoxib. Additionally, incubation with aspirin or celecoxib resulted in the reduction of Bcl‐xL protein level (Figure 8E), suggesting that aspirin may interfere with the survival of senescent cells.

## 4 DISCUSSION

Chemotherapy‐induced long‐term sequelae are a profound concern in childhood cancer survivors.^39^ Although its underlying mechanism is not clearly defined, therapy‐induced accumulation of senescent cells is thought to play a role in the late effects of cancer therapies. In the current study, we hypothesized that the well‐known beneficial effects of NSAIDs against many age‐related diseases may result from the suppression of cellular senescence and, thus, aspirin may suppress chemotherapy‐induced senescence and long‐term adverse effects. To test our hypothesis, we have developed a novel mouse model that mimics the late effects of chemotherapy in the survivors of childhood cancer. Using this mouse model, we have demonstrated that aspirin improves the long‐term adverse effects and suppresses the accumulation of senescent cells in the mice treated with DOX at their juvenile stage.

To our knowledge, there is no mouse model for the late effects of chemotherapy in the survivors of childhood cancer. We used DOX as a chemotherapeutic agent to model the late effects of chemotherapy because anthracyclines including DOX have been widely used to treat children with cancer in the past, suggesting that many current survivors of childhood cancer have received DOX treatment. For example, between 1972 and 1987, children with acute lymphoblastic leukemia were treated with different doses of DOX protocols.^40^ In addition, it is reported that children treated with anthracyclines are at increased risk for various complications.^41^, ^42^, ^43^, ^44^, ^45^ We found that 2‐week‐old mice that received a total of four weekly injections of DOX exhibited various long‐term adverse effects in their adult stage including impaired skeletal growth and reproductive function, fat loss, reduced WBC counts, muscular atrophy, kyphosis, and ocular abnormality. In addition, while DOX has been shown to induce senescence in both adult humans and mice,^5^, ^6^, ^7^ the impact of childhood DOX exposure on senescence in adults has not been determined. We found that the levels of senescence markers are significantly increased in adult mice treated with DOX at their juvenile stage, suggesting that senescent cells may persist and interfere with normal growth and development.

Although numerous human and animal studies have demonstrated aspirin's beneficial effects against various age‐related human diseases, its underlying mechanism remains unclear. Given the close association between senescence and aging, our finding that aspirin suppresses senescence in DOX‐treated mice suggests that aspirin's beneficial effects against age‐related diseases may result from slowing down of the aging process. In support of this, the pro‐longevity effect of NSAIDs including aspirin has been reported,^46^, ^47^, ^48^ although their effects on the health span and underlying mechanisms have not been determined. Our results that aspirin suppresses both the long‐term adverse effects and tissue accumulation of senescent cells in DOX‐treated adult mice further support the hypothesis that the late onset side effects of cancer treatments may result from the abnormal accumulation of senescent cells.

One of the limitations in our study is that we do not know whether the suppression of senescence by aspirin is restricted to DOX or if its beneficial effect can be achieved after other chemotherapy regimens such as antimetabolites or alkylating agents. Anticancer therapies including bleomycin, hydroxyurea, and gamma irradiation have been reported to induce senescence.^49^ Another study has shown that chemotherapeutic agents including paclitaxel, temozolomide, and cisplatin‐induced senescence, indicated by an increase in p16^Ink4a^ promoter‐driven bioluminescence, in p16‐3MR mice.^6^ Given the correlation of cellular senescence and the late effects in our study using DOX, we intend to study whether other senescence‐inducing agents are sufficient to induce late effects in our model system. Future studies will also determine whether senescence induced by these agents can be ameliorated by aspirin.

NSAIDs including aspirin are known to inhibit COX, a rate‐limiting enzyme in the biosynthesis of prostanoids. There are two isoforms of COX: COX1 and COX2. Increasing evidence suggests the role of COX2 in senescence and aging. First, COX2 has been suggested to be involved in the development and/or progression of age‐related human diseases including atherosclerosis, arthritis, cancer, diabetes, osteoporosis, and Alzheimer's disease.^50^, ^51^, ^52^, ^53^, ^54^, ^55^ Second, COX2 expression is increased in the tissues of aged humans and mice. In aged rodents, the increased COX2 expression has been reported in the fat, heart, prostate, liver, kidney, and macrophages.^56^, ^57^, ^58^, ^59^, ^60^, ^61^ The increased COX2 expression has also been reported in the tissues of aged humans, including skin, mononuclear cells, and kidney.^62^, ^63^, ^64^ Third, COX2 expression is induced by virtually all known environmental gerontogens such as arsenic, benzene, cigarette smoke, UV, chemotherapy, and psychological stress,^31^, ^65^, ^66^, ^67^, ^68^, ^69^ whereas caloric restriction, which is known to suppress the aging process, decreases COX2 expression and activity.^70^ Finally, we recently have reported that inducible COX2 expression in adult mice promotes senescence and early aging.^30^

We have previously reported that genetic deletion or pharmacological inhibition of COX2, but not COX1, suppresses DOX‐induced accumulation of p53.^18^ In addition, our current data show that aspirin suppresses DOX‐induced p53 accumulation in normal human fibroblasts and WT MEFs. However, aspirin's suppressive effect on p53 accumulation was significantly reduced in DOX‐treated COX2 KO MEFs. Given the important role of p53 in cellular senescence,^71^ our results suggest that aspirin may interfere with DOX‐induced senescence through the inhibition of COX2. Additionally, our data suggest that aspirin may interfere with the maintenance of senescent cells. Similar to cancer cells, senescent cells make use of various pro‐survival mechanisms to remain viable and rely on antiapoptotic pathways to persist in tissues.^72^ Such mechanisms include, but not limited to, Bcl‐2/Bcl‐xL, PI3K, and p21.^72^, ^73^, ^74^, ^75^ We have found that incubation with aspirin or celecoxib, a COX2‐specific inhibitor, decreases the viability of senescent fibroblasts and reduces the level of Bcl‐xL. Thus, our results suggest that aspirin may affect multiple signaling pathways involved in senescence. It is also possible that aspirin may promote the clearance of senescent cells. Evidence indicates that the senescent cells are removed by the components of the immune system including neutrophils, macrophages, and natural killer cells.^76^ Given the important role of prostaglandin E_2_ (PGE_2_), a major product of COX, in tumor evasion of immune surveillance,^77^, ^78^ it is possible that aspirin may block PGE_2_ synthesis in the senescent cells which are known to express high levels of COX2.^79^ Further studies with inducible COX1 or COX2 KO mice will determine the mechanism by which aspirin suppresses DOX‐induced senescence.

During the preparation of this manuscript, the initial findings from the ASPirin in Reducing Events in the Elderly (ASPREE) trial have been reported, indicating that the daily use of aspirin does not prevent disability‐free survival among older adults but increases bleeding compared with placebo.^80^ Given that this study enrolled people aged 70 and older and that only 11% of participants had regularly taken low‐dose aspirin prior to entering the study, further studies to address aspirin's effects in those who took aspirin longer than 4.7 years in the ASPREE trial are warranted.

In summary, this is the first report showing that aspirin suppresses cellular senescence in vivo. The current data were based on a novel mouse model for the late effects of chemotherapy in the survivors of childhood cancer. Our results support the hypothesis that the late effects of chemotherapy may result from the abnormal accumulation of senescent cells. Our study also provides mechanistic insight into the beneficial effects of aspirin against age‐related diseases. Nonetheless, the mechanism underlying aspirin's effect on cellular senescence is currently unknown. Therefore, further study will be necessary to demonstrate the precise mechanism by which aspirin suppresses chemotherapy‐induced senescence and to determine the contribution of COX isozymes to senescence.

## AUTHOR CONTRIBUTIONS

M Feng performed the research, analyzed the data, and wrote the paper; J Kim, K Field, and C Reid performed the research, analyzed the data, and wrote the paper; I Chatzistamou analyzed the data; M Shim performed the research, designed the study, analyzed the data, and wrote the paper.
