# Supplementary material for: Aspirin ameliorates the long‐term adverse effects of doxorubicin through suppression of cellular senescence
Source: FASEB Bioadv. 2019 Sep 9;1(9):579–90. doi: 10.1096/fba.2019-00041 (PMC6996307; doi:10.1096/fba.2019-00041)
Supplement: Supplementary file 1 [file FBA2-1-579-s001.pdf]

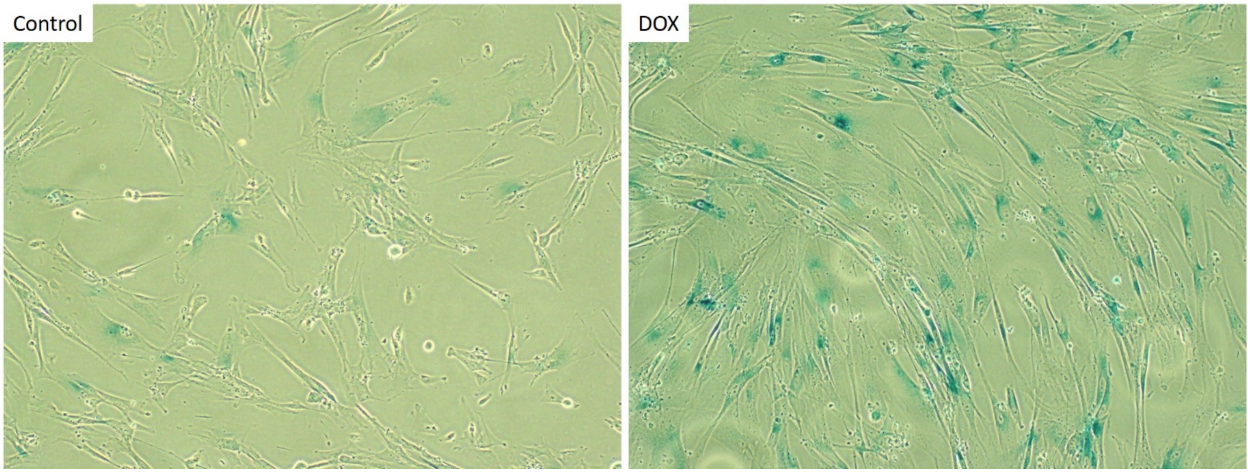

Supplementary figure Senescence-associated β-galactosidase staining of fibroblasts in figure 8D. Senescence was induced by incubation with 0.25 µM DOX for 24 hours. Senescence-associated β-galactosidase staining was conducted at 72 hours after the induction of senescence.
